# Endoscopic Removal of 15 Gastrointestinal Foreign Bodies

**Published:** 2015-09-01

**Authors:** Bircan Savran, Sezgin Zeren, Süleyman Coşgun, Ünal Adigüzel, Ahmet Öztürk, Bercis Imge Ucar

**Affiliations:** 1 Department of Pediatric Surgery, Faculty of Medicine, Dumlupinar University, Kutahya, Turkey; 2 Department of General Surgery, Faculty of Medicine, Dumlupinar University, Kutahya, Turkey; 3 Department of Gastroenterology, Faculty of Medicine, Dumlupinar University, Kutahya, Turkey; 4 Department of Psychiatry, Faculty of Medicine, Dumlupinar University, Kutahya, Turkey

**Keywords:** Foreign body ingestion, Endoscopy, Over-tube, Psychiatric disorder

## Abstract

Foreign body ingestion occurs commonly in children, elderly, mentally impaired or alcoholic, and psychiatric patients. We present a 15-year-old boy with mental retardation and uncontrolled psychiatric disorder admitted to the hospital with abdominal and chest pain. He was diagnosed with foreign body ingestion and 15 foreign objects, including a sharp knife, were successfully removed endoscopically by using an over-tube.

## CASE REPORT

A 15-year-old young male was admitted to the emergency department with abdominal pain radiating to chest and nausea. He had a history of mental retardation and psychiatric disorder, but was not on any medication for these disorders. Slight epigastric tenderness was detected on physical examination. Blood pressure was 125/80 mmHg, oxygen saturation was 93% and other vital findings were normal. Laboratory results were found in normal reference ranges. Numerous foreign bodies were detected in stomach and lower esophagus on plain abdominal radiography (Fig.1).

**Figure:**
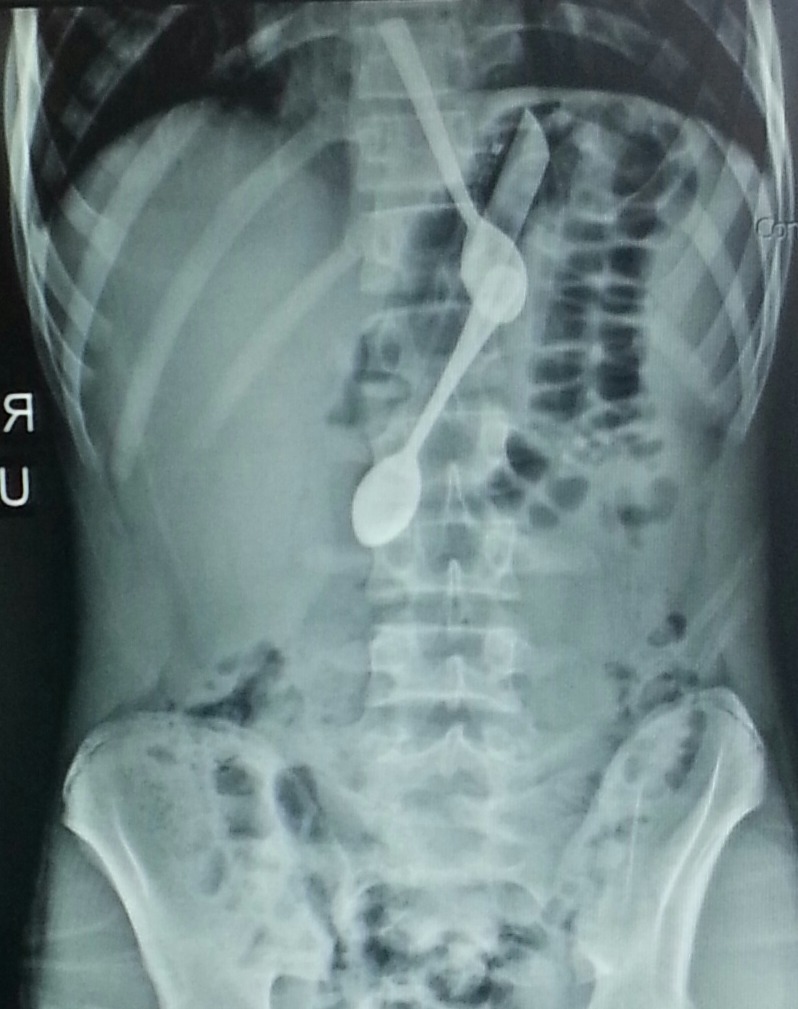
Figure 1:Multiple foreign bodies in the distal part of esophagus and stomach.

Upper flexible GI endoscopy was performed under general anesthesia considering the mental status of the patient. Two tea-spoons, multiple toothpicks, four ice cream sticks, one pencil, one ballpoint pen, one plastic bottle cap, one pipette and one sharp fruit knife were detected in esophagus and stomach (Fig. 2). A total of 15 foreign bodies including the sharp knife were removed endoscopically by using overtube (Fig. 3). The patient allowed orally 6 hour following the endoscopy. He was discharged from the hospital after an overnight stay. Psychiatric follow-up was advised for future surveillance.

**Figure:**
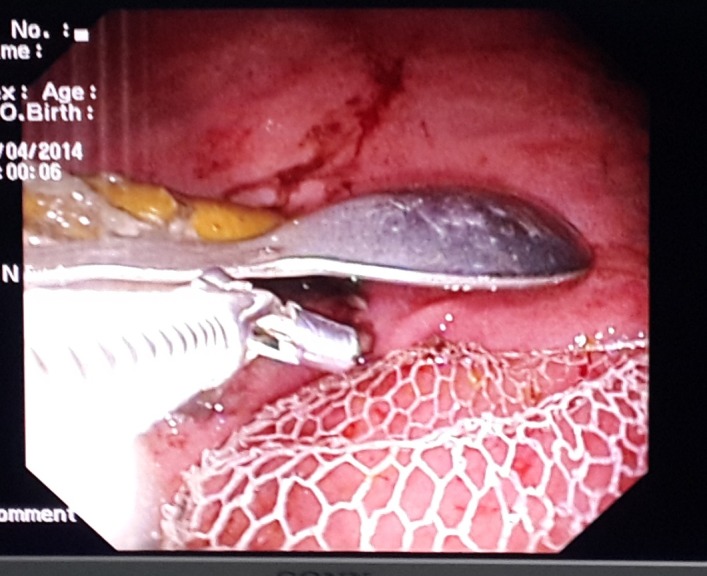
Figure 2:A knife, a teaspoon, a pipette, an ice cream handle, wood pieces, a pencil and a bottle cap can be seen in the endoscopic image of the stomach

**Figure:**
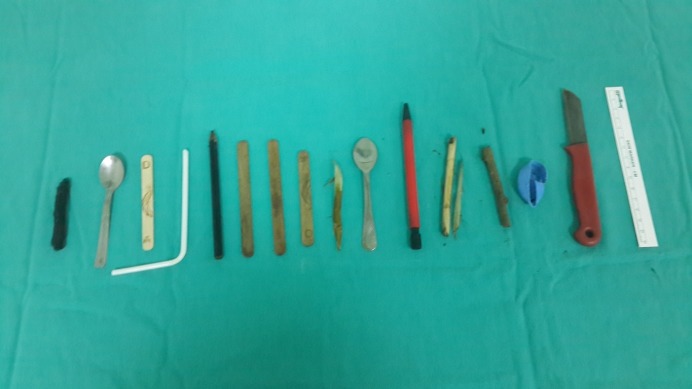
Figure 3:Fifteen foreign bodies that are removed endoscopically from the patients

## DISCUSSION

Ingestion of foreign bodies into gastrointestinal (GI) tract is one of the common causes of admission to emergency departments.[1] The problem is encountered in all age groups; however, nearly 80% of the cases comprise children who swallowed coins, toys, etc. It also occurs in those with psychiatric problems, behavioral disorders, mental retardation, etc.[2,3] Most of the ingested foreign bodies pass through the digestive tract spontaneously without requiring any treatment. About 10-20% of the patients need endoscopic management and less than 1% undergoes surgery [3]. Retained esophageal foreign bodies can lead to significant complications, including stricture formation, esophageal perforation, tracheoesophageal and aorto-esophageal fistula etc.[4]

Fiberoptic or rigid endoscopy are used to diagnose and also for removal of foreign bodies.[5] In the present case, flexible endoscopy was preferred since the foreign bodies were localized in distal esophagus and stomach. Complications such as perforation, bleeding, mucosal injury, may occur during extraction of these objects endoscopically.[5] We used over-tube in order to protect the GI mucosa from trauma, limit the risk of aspiration, increase the depth of insertion, and maintain access for repeated withdrawal and reinsertion; and successfully removed all foreign bodies including a sharp knife.

## Footnotes

**Source of Support:** Nil

**Conflict of Interest:** None declared

